# Supplementary material for: Using phenotyping to visualize and identify selfish bacteria: a methods guide
Source: Microbiol Spectr. 2025 Jul 7;13(8):e01602-24. doi: 10.1128/spectrum.01602-24 (PMC12323593; doi:10.1128/spectrum.01602-24)
Supplement: Supplemental figures and tables — Figures S1 to S5 and Tables S1 to S4. [file spectrum.01602-24-s0001.pdf]

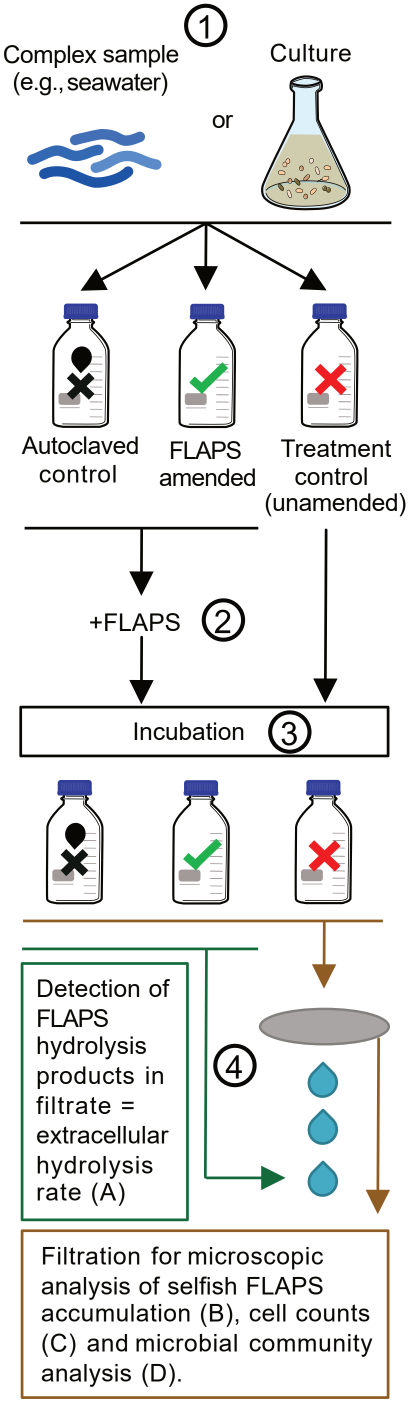

Supplementary Figure S1 Workflow to analyze extracellular polysaccharide hydrolysis **(A)**, selfish polysaccharide uptake **(B)**, total cell counts **(C)** pure cultures and the underlying microbial community **(D)** from complex samples. **1** Priming with unlabeled polysaccharides of pure cultures show the general potential to utilize polysaccharides **2** Low or ambient concentrations testing for the natural potential to utilize polysaccharides. High concentrations to show the general potential to utilize polysaccharides. **3** Long incubation (days - weeks) testing for the natural potential to utilize polysaccharides in natural waters. Short incubation (hours - days) to show the general potential to utilize polysaccharides or the natural potential to use polysaccharides in sediments or other cell-rich natural environments. **4** Fixation of the sample might be required for further analysis. Formaldehyde fixation, for example, stabilizes the cell structure, stabilizes the substrate in the cell and maintains morphology for microscopic analysis.

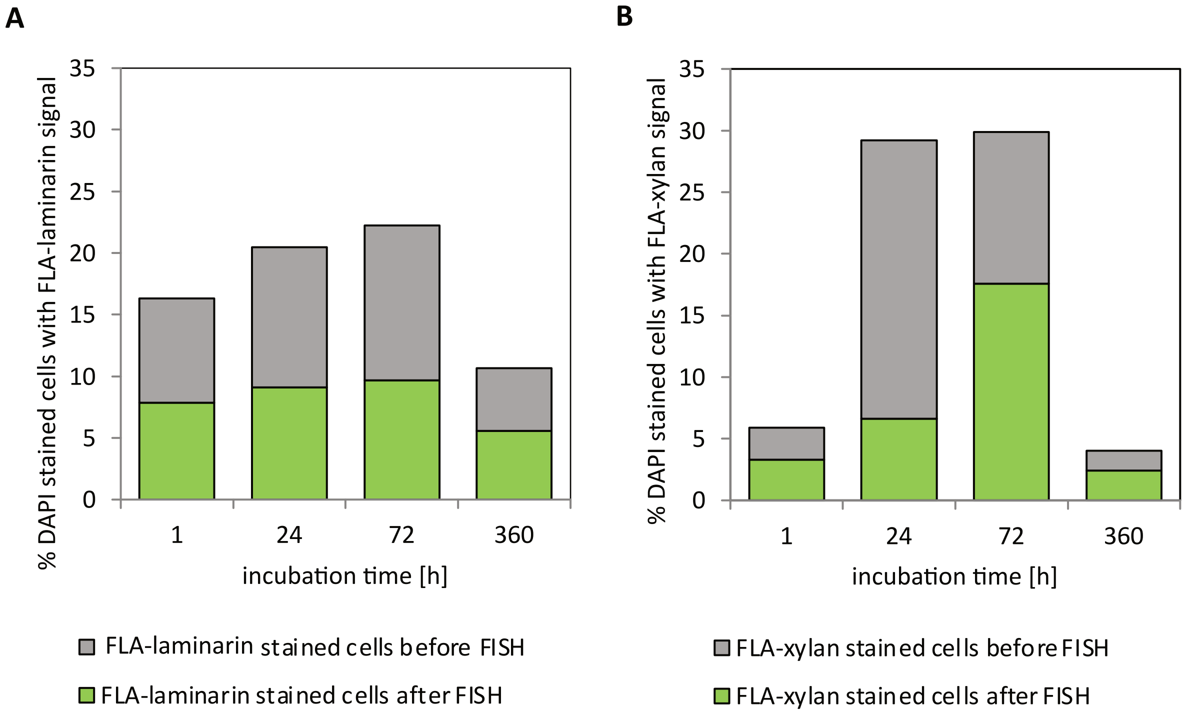

Supplementary Figure S2 Proportion of **(A)** FLA-laminarin stained cells and **(B)** FLA-xylan stained cells before and after FISH treatment.

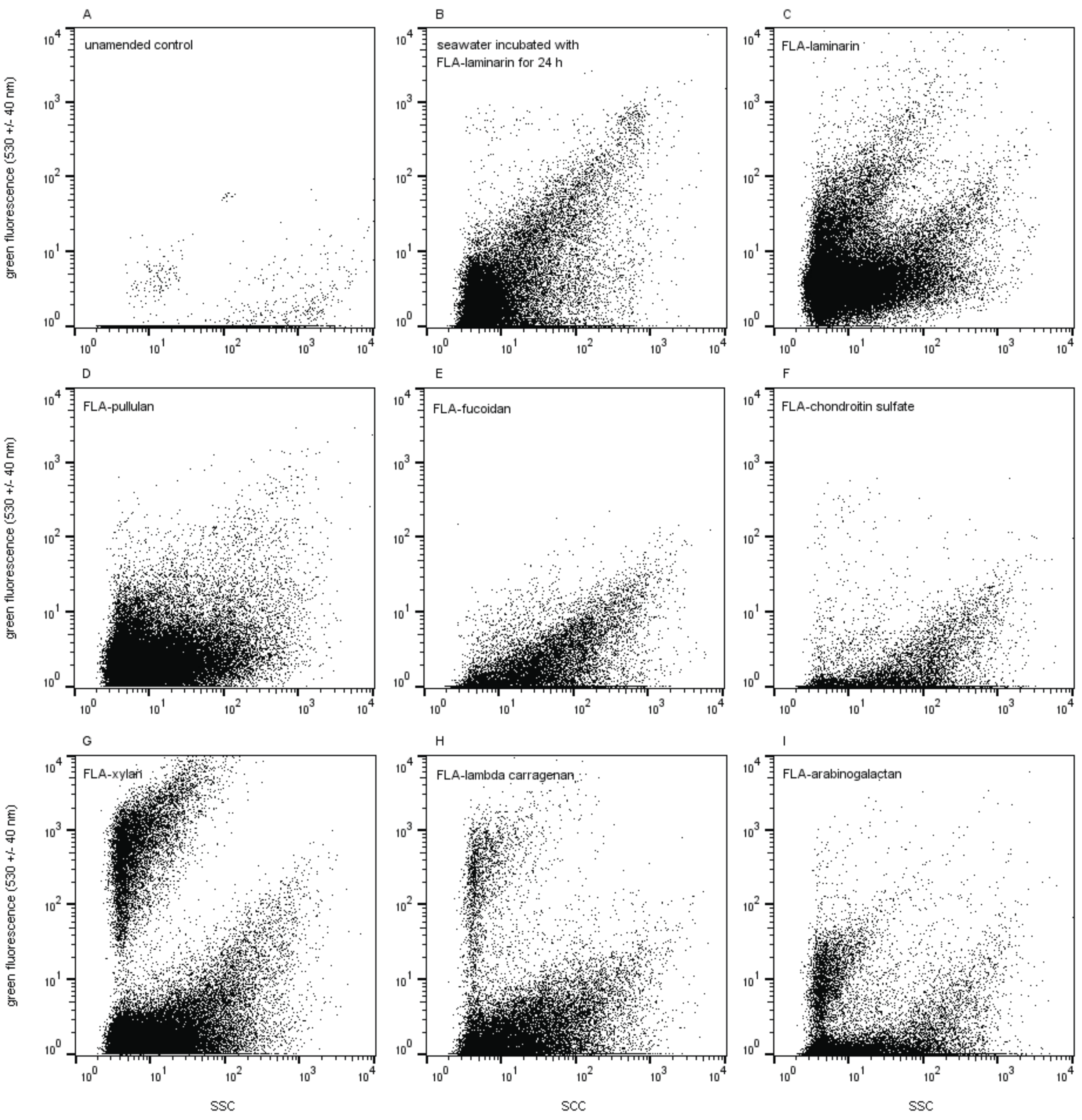

Supplementary Figure S3 Flow cytometric dot plots showing FLAPS background signals in marine surface waters. **(A)** Seawater without the addition of any FLAPS to define the samples background noise. **(B)** Seawater sample containing some FLA-laminarin stained cells after 24 h of incubation. Formamide fixed seawater supplemented with **(C)** FLA-laminarin, **(D)** FLA-pullulan, **(E)** FLA-fucoidan, **(F)** FLA-chondroitin sulfate, **(G)** FLA-xylan, **(H)** FLA-lambda carrageenan and **(I)** FLA-arabinogalactan to define substrate background noise.

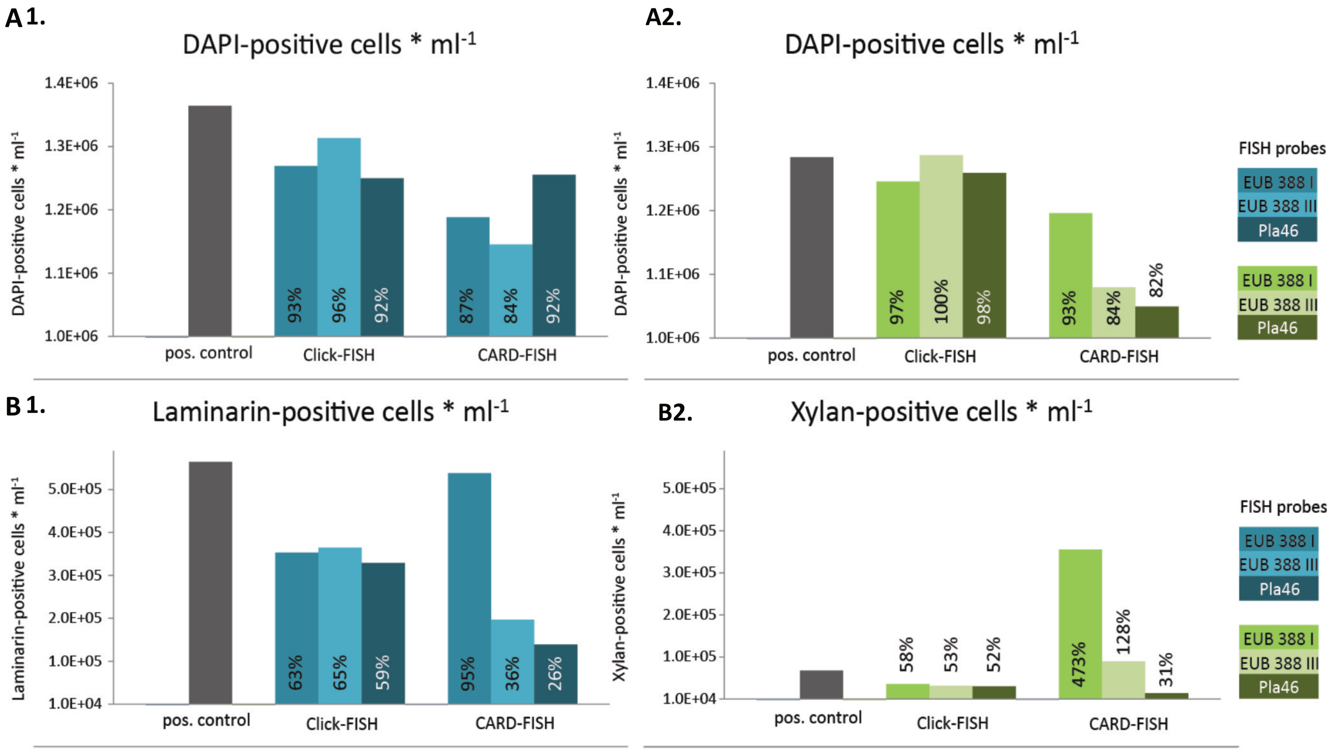

Supplementary Figure S4 Comparison of FLA-substrate labeled cells from summer incubations of Helgoland seawater, taxonomically stained using tetra-labeled FISH probes and CARD-FISH; **(A1&2)** DAPI-positive cells without treatment (positive control (pos. control)) and after FISH treatment in the 1) laminarin and 2) xylan incubations. The percentages of DAPI-positive counts after FISH treatment relates to total DAPI-positive cells in pos. control. **(B1&2)** Substrate-positive cells without treatment (pos. control) and after FISH treatment in the 1) laminarin and 2) xylan incubations. The percentages of substrate-positive counts after FISH treatment relates to total substrate-positive cells in pos. control

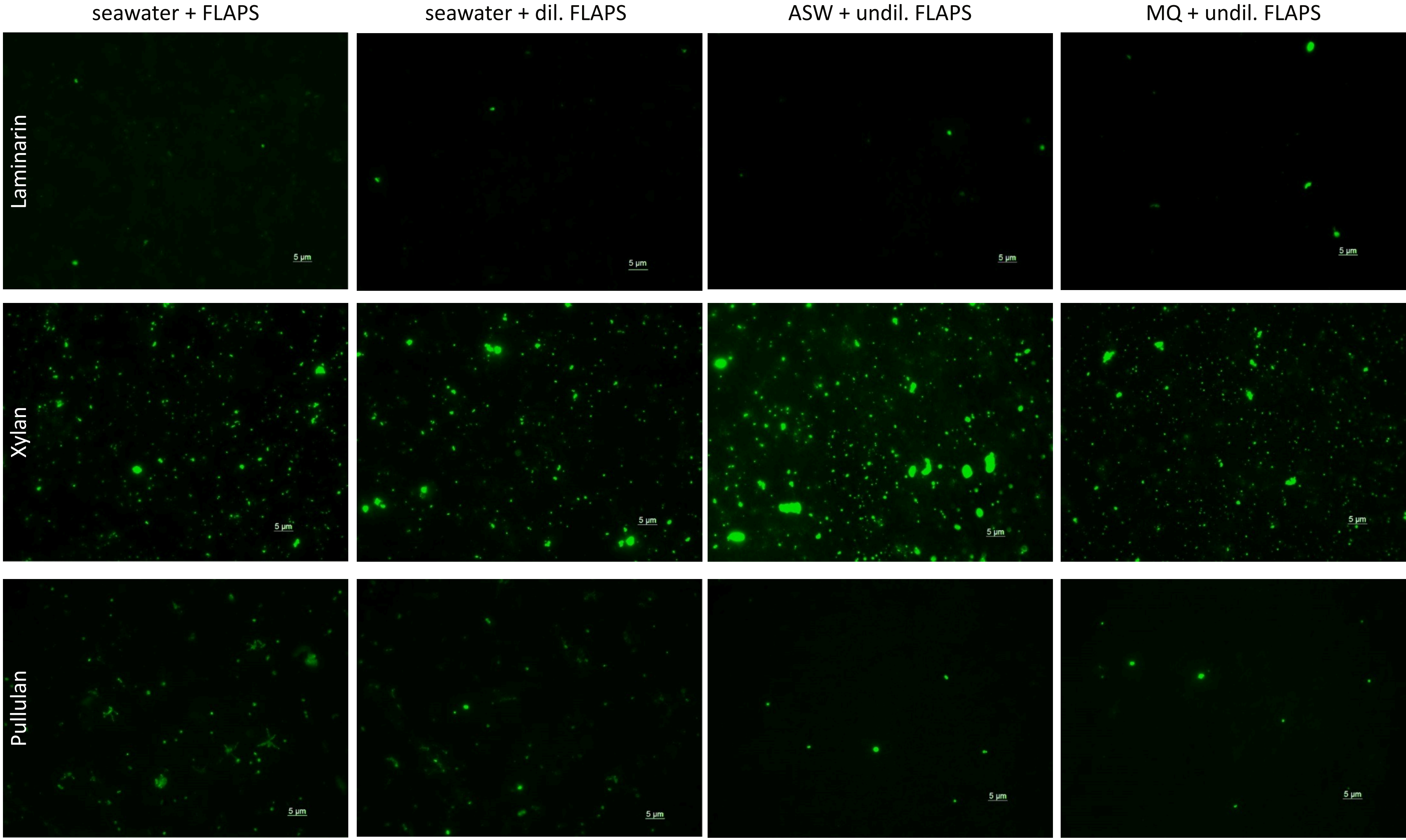

Supplementary Figure S5 Comparison of FLAPS and DAPI staining is required to identify substrate-stained cells. The background noise of fluorescently labeled polysaccharides (FLA-PS) after substrate dilution in different solvents. ASW = artificial seawater, MQ = 18 MΩcm water.

Supplementary Table S1 Polysaccharides that have been fluorescently labelled for the extracellular enzyme (EE) or selfish uptake analysis. Highlighted are the habitats and organisms in which these polysaccharides have been tested. Note that studies carried out prior to 2012 did not in any case include investigation of selfish uptake. See Arnosti et al. 2011 for a partial summary of prior investigations in marine planktonic environments.

Polysaccharide	Sugar Type	Monosaccharide units	Habitat	Bacterial culture	Extracellular hydroylsis (EE) /Selfish	Reference*
Alginic acid	1,4-glycosidic bond	β-D-mannuronate and α-L-guluronate	Seawater, (Arctic) oxic and anoxic marine sediment		EE	Arnosti, 2008, Teske et., al. 2011
Arabinogalactan	β-1,3, β-1,5, β-1,6 (Galactan), α-1,3, α-1,5,				EE	Arnosti 2000; 2008 Arnosti et al. 2009; 2011a; Ziervogel & Arnosti 2009; Teske et al. 2011; Steen et al. 2012; Arnosti & Steen 2013; Cardman et al. 2014; D'Ambrosio et al. 2014; Steen & Arnosti 2014; Bullock et al. 2015; Hoarfrost & Arnosti 2017; Balmonte et al., 2018; 2019; 2021; 2024; Hoarfrost et al. 2019; Brown et al. 2022; Lloyd et al. 2022; 2023; 2025;
			Seawater (Estuarine, coastal, temperate, polar and gyre regions, marine particles)	Gramella forsetti	EE / Selfish	
			Aggregates and seawater		EE	
			Oxic and anoxic marine sediment		EE	Arnosti 2000; Arnosti & Holmer, 2003; Arnosti et al. 2009; Hubert et al. 2010; Teske et al. 2011; Cardman et al. 2014
			Freshwater riverine and limnic		EE / Selfish	Cackiovic et. al., 2025; EE: Balmonte et al. 2016
Amylopectin	α-1,4	glucose		Bacteroidetes thetaiotaomicron	Selfish	unpublished
Chondrotin Sulfate		N-acetylgalactosamine, glucoronic acid	Seawater (Coastal, temperate, polar and gyre regions), marine particles		EE / Selfish	EE: Arnosti 2000; 2008 Arnosti et al. 2005; 2009; 2011a,b; 2012; Ziervogel & Arnosti 2009; Teske et al. 2011; Steen et al 2012; Arnosti & Steen 2013; Cardman et al. 2014; D'Ambrosio et al. 2014; Stehen & Arnosti 2014; Bullock et al. 2015; Hoarfrost & Arnosti 2017; Balmonte et al., 2018; 2019a,b; 2021; 2024; Hoarfrost et al. 2019; Brown et al. 2022; Lloyd et al. 2022; 2023; 2025;<br><br>Reintjes et al., 2017; 2019; 2020a,b; Manna et al., 2022; Giljan et al. 2022; 2023; Brown et al. 2024; Knittel et. al., 2024
			Aggregates and seawater		EE	Ziervogel & Arnosti 2008; 2016; Ziervogel et al. 2010; 2016; 2019; Arnosti et al. 2016
			Oxic and anoxic marine sediment		EE	
			Freshwater riverine and limnic		EE / Selfish	Cackiovic et. al., 2025, Steen et al. 2008; Ziervogel et al. 2014; Bullock et al. 2017
Direct microalgae extract	Diverse	Diverse	sediments		EE	Arnosti et al. 2005; Teske et al. 2011
Isochrysis			Seawater, mesocosm<br>Sewater & sediments		EE	Murry et al., 2007<br>Arnosti 2008; Arnosti et al. 2009
Skeletonema sp.			Seawater, mesocosm		EE	Murry et al., 2007; Arnosti et al. 2009
Spirulina			Seawater & sediments		EE	Arnosti 2008; Teske et al. 2011
Direct macroalgae extract	Diverse	Diverse				
Saccharina latissima				Bacteroidetes thetaiotaomicron	Selfish	Ferrillo et al., 2024
Ascophyllum nodosum			Anaerobic rumen and artifical rumen system	Bacteroidetes thetaiotaomicron	Selfish	unpublished
Fucoidan	Diverse	fucose (galactose, xylose, arabinose, rhamnose)	Seawater (Coastal, temperate, polar and gyre regions, marine particles)		EE / Selfish	EE: Arnosti 2000; 2008; Arnosti et al. 2005; 2009; 2011a,b; 2012; Ziervogel & Arnosti 2009; Teske et al. 2011; Steen et al. 2012; Arnosti & Steen 2013; Cardman et al. 2014; D'Ambrosio et al. 2014; Steen & Arnosti 2014; Bullock et al. 2015; Hoarfrost & Arnosti 2017; Balmonte et al., 2018; 2019a,b; 2021; 2024; Hoarfrost et al. 2019; Brown et al. 2022; Lloyd et al. 2022; 2023; 2025;<br><br>Reintjes et al., 2017; 2019; 2020b, Giljan et al. 2023; Brown et al. 2024; Knittel et. al., 2024
			Aggregates and Seawater		EE	Ziervogel & Arnosti 2008; 2016; 2020; Ziervogel et al. 2010; 2016; 2019; Arnosti et al. 2016
			Oxic and anoxic marine sediment		EE	Arnosti, 2000; 2003, 2008; Arnosti & Jørgensen 2003; 2006; Arnosti & Holmer 2003; Arnosti et al. 2005; 2009; Hubert et al. 2010; Teske et al. 2011; Cardman et al. 2014
			Freshwater (limnic)		EE / Selfish	Cackiovic et. al., 2025; EE: Keith & Arnosti 2001,Steen et al. 2008; Ziervogel et al. 2014; Bullock et al. 2017
Glycogen	α-1,4. α-1,6	Glucose		Bacteroidetes thetaiotaomicron	Selfish	unpublished
				Marine bacteria isolates	Selfish	in prep. Reintjes Lab, Uni Bremen
Inulin	β-2,1	Fructose		Rumen Bifidobacterium and Lactobacillus species	Selfish	King et.al., 2024
Laminarin		Glucose		Gramella forsetti	EE / Selfish	EE: Arnosti 2000; 2008; Arnosti et al. 2005; 2009; 2011a,b; 2012; Ziervogel & Arnosti 2009; Teske et al. 2011; Steen et al. 2012; Arnosti & Steen 2013; Cardman et al. 2014; D'Ambrosio et al. 2014; Steen & Arnosti 2014; Bullock et al. 2015; Hoarfrost & Arnosti 2017; Balmonte et al., 2018; 2019a,b; 2021; 2024; Hoarfrost et al. 2019; Brown et al. 2022; Lloyd et al. 2022; 2023; 2025; Moncada et al. 2024<br><br>Reintjes et al., 2017; 2019; 2020a,b, Manna et al., 2022; Giljan et al. 2022; 2023; Brown et al. 2024; Knittel et. al., 2024
			Aggregates and Seawater		EE	Ziervogel & Arnosti 2008; 2016; 2020; Ziervogel et al. 2010; 2016; 2019; Arnosti et al. 2016
			Oxic and anoxic marine sediment		EE	Arnosti, 1996; 1998; 2000; 2003, 2008; Arnosti & Holmer 1999; 2003; Arnosti & Jørgensen 2006; Boer et al. 2009; Arnosti et al. 2009; Julies et al. 2010; Teske et al. 2011; Hoarfrost et al.
			Freshwater riverine & limnic		EE / Selfish	Cackiovic et. al., 2025; EE: Keith & Arnosti 2001; Steen et al. 2008; Ziervogel et al. 2014; Bullock et al. 2017
Levan	β-2,6	Fructose		Rumen Bifidobacterium and Lactobacillus species	Selfish	King et.al., 2024
Mucin	Diverse	Diverse	Seawater		Selfish / EE	Knittel et. al., 2024
			Marine sediment		Selfish / EE	Knittel et. al., 2024
Porphyran	β-1,3, α-1,4	galactosyl, galactosyl 6-sulfate or 3,6-anhydrogalactosyl		Bacteroides plebeius, Bacteroides uniformis, Bacteroidetes thetaiotaomicron, Bacteroidetes ovatus	Selfish	Robb et al., 2022
Pullulan	α-1,4, α-1,6	Glucose	Seawater (Coastal, temperate, polar, and gyre regions),	Gramella forsetti	EE	Arnosti 2000; 2008; Arnosti et al. 2005; 2009; Ziervogel & Arnosti 2009; Teske et al. 2011; Arnosti et al. 2011a; Steen et al. 2012; Arnosti & Steen 2013; Cardman et al. 2014; D'Ambrosio et al. 2014; Steen & Arnosti 2014; Bullock et al. 2015; Hoarfrost & Arnosti 2017; Balmonte et al., 2018; 2019a,b; 2021; 2024; Hoarfrost et al. 2019; Brown et al. 2022;
			marine particles		Selfish / EE	Reintjes et al., 2017; 2019; 2020b, Manna et al., 2022; Giljan et al. 2023; Brown et al. 2024
			Aggregates and Seawater		EE	Ziervogel & Arnosti 2008; 2016; 2020; Ziervogel et al. 2010; 2016; 2019; Arnosti et al. 2016
			Oxic and anoxic marine sediment		EE	Arnosti, 1995; 1996; 1998; 2000; 2003, 2008; Arnosti et al. 1998; Arnosti & Holmer 1999; 2003 Arnosti & Jørgensen 2003; 2006; Arnosti et al. 2005; 2009; Hubert et al. 2010; Julies et al. 2010; Teske et al. 2011; Cardman et al. 2014
			Freshwater riverine & limnic		EE / Selfish	Cackiovic et. al., 2025, Keith & Arnosti 2001, Balmonte et al. 2016
Rhamnogalacturonan II	diverse	Homogalacturan, galacturonic acid, galactose, rhamnose, arabinose, galactan		Bacteroidetes thetaiotaomicron	Selfish	Hehemann et al., 2019
Xylan			Seawater (Coastal, temperate, polar, and gyre regions), marine particles		Selfish / EE	EE: Arnosti, 2000; 2008; Arnosti et al. 2005; 2009; 2011a,b; 2012; Murray et al. 2007; Ziervogel &Arnosti 2009; Teske et al. 2011; Steen et al. 2012; Arnosti & Steen 2013; Cardman et al. 2014; D'Ambrosio et al. 2014; Steen & Arnosti 2014; Bullock et al. 2015; Hoarfrost & Arnosti 2017; Balmonte et al. 2018; 2019a,b; 2021; 2024; Hoarfrost et al. 2019; Brown et al. 2022; Lloyd et al. 2022; 2023; 2025. EE/Selfish: Reintjes et al., 2017; 2019; 2020a,b, Manna et al., 2022; Giljan et al. 2022; 2023; Brown et al. 2024; Knittel et. al., 2024
			Aggregates and seawater		EE	Ziervogel & Arnosti 2008; 2016; 2020; Ziervogel et al. 2010; 2016; 2019; Arnosti et al. 2016
			Oxic and anoxic marine sediment		EE	Arnosti 1998; 2000; 2003, 2008; Arnosti & Holmer 2003; Arnosti & Jørgensen 2003; 2006; Arnost et al. 2005; 2009; Teske et al. 2011; Cardman et al. 2014; Lloyd et al. 2025
			Freshwater, riverine & limnic		EE / Selfish	Selfish: Cackiovic et. al., 2025; EE: Keith & Arnosti 2001, Steen et al. 2008; Ziervogel et al. 2014; Bullock et al. 2017
Yeast-mannan	α-1,6 (backbone), α-1,3, α-1,2 (side chains)	Mannose	Seawater; Anaerobic rumen and mouse gastrointestinal tract	Bacteroidetes thetaiotaomicron	Selfish/EE	selfish: Hehemann et al., 2019, Klassen et al., 2021;Selfish/EE: Lloyd et al. (in prep)
λ-carrageenan	β-1,3, α-1,4	galactopyranose, 3,6-anhydrogalactopyranose	Seawater (Coastal, temperate and gyre regions)		Selfish / EE	Reintjes et al., 2020

* see extended reference for Supplementary Table S1: Reference list of all cited publications in supplementary Table S1.

Supplementary Table S2 Recommended settings for the evaluation of FLA-polysaccharide stained cells and their taxonomic identification by fluorescence *in situ* hybridization with an automated epifluorescence microscope and the image analysis software ACMETool; 1 pixel = 0.10601 µm^2. SBR = signal-to-background ration; MGV = medium grey value.

Dye	Excitation wavelength [nm]	Exposure time [ms]	Signal definition (ACMETool)		
			Area [pixel]	SBR	MGV
4′,6-Diamidin-2-phenylindol (DNA stain, UV)	365 ± 4.5	25	18 – 150	2	55
Fluoresceinamine (FLA-polysaccharide)	470 ± 14	140<br>35<br>10	18 – 200<br>18 – 250<br>18 – 200	1.8<br>2<br>2	55<br>65<br>55
4xAtto594 (Mono-FISH)	590 ± 17.5	110	18 – 400	2	55
Atto594 (CARD-FISH)	590 ± 17.5	70	18 – 400	2	55
Auto fluorescence	590 ± 17.5	500	>6		

Supplementary Table 3 Fluorescence *in situ* hybridization probe overview.

Probe	Target organisms	Sequence (5' -> 3')	Formamide [%]	Reference
EUB338-I	Bacteria	GCTGCCTCCCGTAGGAGT	35	Amann *et al.,* 1990
EUB338-III	*Verrucomicrobia*	GCTGCCACCCGTAGGTGT	35	Daims *et al.,* 1999
PLA46	*Planctomycetes*	GACTTGCATGCCTAATCC	30	Neef *et al.,* 1998
CF319a	*Bacteroidetes*	TGGTCCGTGTCTCAGTAC	35	Manz *et al.,* 1996
GAM42a	*Gammaproteobacteria*	GCCTTCCCACATCGTTT	35	Manz *et al.,* 1992

Supplementary Table S4 Treatment overview for substrate background signal testing. FOV = field of view;ASW = artifical seawater; MQ = 18 MΩcm water; FA = formaldehyde; FLAPS = fluorescently labeled polysaccharide; EDTA = Ethylenediaminetetraacetic acid; - = not tested; N.D. = not detectible due to overexposure.

		Background signal counts at 35 ms/ FOV					
	Treatment: FLAPS + [X]	Laminarin	Xylan	Chondroitin sulfate	Pullulan	Arabino-glactan	Fucoidan
A	X = seawater + 1% FA	23	1553	9	316	12	2
B	X = ASW + 1% FA	166	N.D.	82	N.D.	145	216
C	X = MQ + 1% FA	N.D.	N.D.	725	N.D.	527	N.D.
D	X = ASW	18	1078	3	86	8	2
E	X = MQ	17	1130	10	720	5	3
F	X = seawater + 1% FA, FLAPS = 100 x dilluted	42	1112	5	204	10	1
G	X = seawater + 25 mMol EDTA	N.D.	N.D.	N.D.	-	-	-
H	X = ASW + 25 mMol EDTA	N.D.	N.D.	N.D.	-	-	-
I	X = MQ + 25 mMol EDTA	91	1195	143	-	-	-
J	X = ASW + 5 min at 32 °C prewarmed FLAPS	26	1630	N.D.	-	-	-
K	X = ASW + 15 min at 32 °C prewarmed FLAPS	321	1403	48	-	-	-
L	X = MQ + 5 min at 32 °C prewarmed FLAPS	104	1847	446	-	-	-
M	X = MQ + 15 min at 32 °C prewarmed FLAPS	93	1591	499	-	-	-
N	X = seawater + 5 min at 32 °C prewarmed FLAPS	405	1743	N.D.	-	-	-
O	X = seawater + 15 min at 32 °C prewarmed FLAPS	215	1525	N.D.	-	-	-
