# Supplementary material for: Using phenotyping to visualize and identify selfish bacteria: a methods guide
Source: Microbiol Spectr. 2025 Jul 7;13(8):e01602-24. doi: 10.1128/spectrum.01602-24 (PMC12323593; doi:10.1128/spectrum.01602-24)
Supplement: Supplemental material — Reference list of all cited publications in Table S1. [file spectrum.01602-24-s0002.docx]

Extended reference for Supplementary Table S1: Reference list of all cited publications in supplementary Table S1.

1. Arnosti C. 1996. A new method for measuring polysaccharide hydrolysis rates in marine environments. Organic Geochemistry 25:105-115.1998
2. Arnosti C. 1998. Rapid potential rates of extracellular enzymatic hydrolysis in Arctic sediments. Limnology and Oceanography 43:315-324.
3. Arnosti C. 2000. Substrate specificity in polysaccharide hydrolysis: Contrasts between bottom water and sediments. Limnology and Oceanography 45:1112-1119.
4. Keith SC, Arnosti C. 2001. Extracellular enzyme activity in a river-bay-shelf transect: variations in polysaccharide hydrolysis rates with substrate and size class. Aquatic Microbial Ecology 24:243-253.2003
5. Arnosti C. 2003. Fluorescent derivatization of polysaccharides and carbohydrate-containing biopolymers for measurement of enzyme activities in complex media. Journal of Chromatography B 793:181-191.
6. Arnosti C, Holmer M. 2003. Carbon cycling in a continental margin sediment: contrasts between organic matter characteristics and remineralization rates and pathways. Estuarine, Coastal and Shelf Science 58:197-208.
7. Arnosti C, Jørgensen BB. 2003. High activity and low temperature optima of extracellular enzymes in Arctic sediments: implications for carbon cycling by heterotrophic microbial communities. Marine Ecology Progress Series 249:15-24.
8. Arnosti C, Durkin S, Jeffrey WH. 2005. Patterns of extracellular enzyme activities among pelagic marine microbial communities: implications for cycling of dissolved organic carbon. Aquatic Microbial Ecology 38:135-145.
9. Arnosti C, Jørgensen BB. 2006. Organic Carbon Degradation in Arctic Marine Sediments, Svalbard: A Comparison of Initial and Terminal Steps. Geomicrobiology Journal 23:551-563.
10. Murray AE, Arnosti C, De La Rocha CL, Grossart HP, Passow U. 2007. Microbial dynamics in autotrophic and heterotrophic seawater mesocosms. II. Bacterioplankton community structure and hydrolytic enzyme activities. Aquatic Microbial Ecology 49:123-141.
11. Arnosti C. 2008. Functional differences between Arctic seawater and sedimentary microbial communities: contrasts in microbial hydrolysis of complex substrates. FEMS Microbiology Ecology 66:343-351.
12. Ziervogel K, Arnosti C. 2008. Polysaccharide hydrolysis in aggregates and free enzyme activity in aggregate-free seawater from the north-eastern Gulf of Mexico. Environ Microbiol 10:289-99.
13. Steen AD, Hamdan LJ, Arnosti C. 2008. Dynamics of dissolved carbohydrates in the Chesapeake Bay: Insights from enzyme activities, concentrations, and microbial metabolism. Limnology and Oceanography 53:936-947.
14. Arnosti C, Ziervogel K, Ocampo L, Ghobrial S. 2009. Enzyme activities in the water column and in shallow permeable sediments from the northeastern Gulf of Mexico. Estuarine, Coastal and Shelf Science 84:202-208.
15. Ziervogel K, Arnosti C. 2009. Enzyme activities in the Delaware Estuary affected by elevated suspended sediment load. Estuarine, Coastal and Shelf Science 84:253-258.
16. Böer SI, Arnosti C, van Beusekom JEE, Boetius A. 2009. Temporal variations in microbial activities and carbon turnover in subtidal sandy sediments. Biogeosciences 6:1149-1165.
17. Hubert C, Arnosti C, Brüchert V, Loy A, Vandieken V, Jørgensen BB. 2010. Thermophilic anaerobes in Arctic marine sediments induced to mineralize complex organic matter at high temperature. Environmental Microbiology 12:1089-1104.
18. Ziervogel K, Steen AD, Arnosti C. 2010. Changes in the spectrum and rates of extracellular enzyme activities in seawater following aggregate formation. Biogeosciences 7:1007-1015.
19. Julies EM, Fuchs BM, Arnosti C, Brüchert V. 2010. Organic Carbon Degradation in Anoxic Organic-Rich Shelf Sediments: Biogeochemical Rates and Microbial Abundance. Geomicrobiology Journal 27:303-314.
20. Teske A, Durbin A, Ziervogel K, Cox C, Arnosti C. 2011. Microbial Community Composition and Function in Permanently Cold Seawater and Sediments from an Arctic Fjord of Svalbard. Applied and Environmental Microbiology 77:2008-2018.
21. Arnosti C. 2011. Microbial extracellular enzymes and the marine carbon cycle. Annual Review of Marine Science 3:401-425.
22. Arnosti C, Steen AD, Ziervogel K, Ghobrial S, Jeffrey WH. 2011. Latitudinal Gradients in Degradation of Marine Dissolved Organic Carbon. PLoS ONE 6:e28900.
23. Steen AD, Ziervogel K, Ghobrial S, Arnosti C. 2012. Functional variation among polysaccharide-hydrolyzing microbial communities in the Gulf of Mexico. Marine Chemistry 138:13-20.
24. Arnosti C, Fuchs BM, Amann R, Passow U. 2012. Contrasting extracellular enzyme activities of particle-associated bacteria from distinct provinces of the North Atlantic Ocean. Frontiers in microbiology 3:425.
25. Arnosti C, Steen AD. 2013. Patterns of extracellular enzyme activities and microbial metabolism in an Arctic fjord of Svalbard and in the northern Gulf of Mexico: contrasts in carbon processing by pelagic microbial communities. Frontiers in microbiology 4:318.2014
26. Cardman Z, Arnosti C, Durbin A, Ziervogel K, Cox C, Steen AD, Teske A. 2014. *Verrucomicrobia* are candidates for polysaccharide-degrading bacterioplankton in an arctic fjord of Svalbard. Applied and Environmental Microbiology 80:3749-3756.
27. D'Ambrosio L, Ziervogel K, MacGregor B, Teske A, Arnosti C. 2014. Composition and enzymatic function of particle-associated and free-living bacteria: a coastal/offshore comparison. The ISME Journal 8:2167-2179.•
28. Steen AD, Arnosti C. 2014. Picky, hungry eaters in the cold: persistent substrate selectivity among polar pelagic microbial communities. Frontiers in microbiology 5:527.
29. Ziervogel K, Leech D, Arnosti C. 2014. Differences in the substrate spectrum of extracellular enzymes in shallow lakes of differing trophic status. Biogeochemistry 117:143-151.
30. Bullock A, Ziervogel K, Ghobrial S, Jalowska A, Arnosti C. 2015. Microbial activities and organic matter degradation at three sites in the coastal North Atlantic: Variations in DOC turnover times and potential for export off the shelf. Marine Chemistry 177:388-397.
31. Arnosti C, Ziervogel K, Yang T, Teske A. 2016. Oil-derived marine aggregates – hot spots of polysaccharide degradation by specialized bacterial communities. Deep Sea Research Part II: Topical Studies in Oceanography 129:179-186.
32. Ziervogel K, Dike C, Asper V, Montoya J, Battles J, D׳souza N, Passow U, Diercks A, Esch M, Joye S, Dewald C, Arnosti C. 2016. Enhanced particle fluxes and heterotrophic bacterial activities in Gulf of Mexico bottom waters following storm-induced sediment resuspension. Deep Sea Research Part II: Topical Studies in Oceanography 129:77-88.
33. Balmonte JP, Arnosti C, Underwood S, McKee BA, Teske A. 2016. Riverine Bacterial Communities Reveal Environmental Disturbance Signatures within the Betaproteobacteria and Verrucomicrobia. Frontiers in Microbiology 7.
34. Hoarfrost A, Arnosti C. 2017. Heterotrophic Extracellular Enzymatic Activities in the Atlantic Ocean Follow Patterns Across Spatial and Depth Regimes. Frontiers in Marine Science 4.
35. Reintjes G, Arnosti C, Fuchs BM, Amann R. 2017. An alternative polysaccharide uptake mechanism of marine bacteria. ISME J 11:1640-1650.
36. Bullock A, Ziervogel K, Ghobrial S, Smith S, McKee B, Arnosti C. 2017. A Multi-season Investigation of Microbial Extracellular Enzyme Activities in Two Temperate Coastal North Carolina Rivers: Evidence of Spatial but Not Seasonal Patterns. Frontiers in Microbiology 8.
37. Balmonte JP, Teske A, Arnosti C. 2018. Structure and function of high Arctic pelagic, particle-associated and benthic bacterial communities. Environmental Microbiology 20:2941-2954.
38. Balmonte JP, Buckley A, Hoarfrost A, Ghobrial S, Ziervogel K, Teske A, Arnosti C. 2019. Community structural differences shape microbial responses to high molecular weight organic matter. Environmental Microbiology 21:557-571.
39. Hoarfrost A, Balmonte JP, Ghobrial S, Ziervogel K, Bane J, Gawarkiewicz G, Arnosti C. 2019. Gulf Stream Ring Water Intrusion on the Mid-Atlantic Bight Continental Shelf Break Affects Microbially Driven Carbon Cycling. Frontiers in Marine Science 6.
40. Reintjes G, Arnosti C, Fuchs B, Amann R. 2019. Selfish, sharing and scavenging bacteria in the Atlantic Ocean: a biogeographical study of bacterial substrate utilisation. ISME J 13:1119-1132.
41. Hehemann JH, Reintjes G, Klassen L, Smith AD, Ndeh D, Arnosti C, Amann R, Abbott DW. 2019. Single cell fluorescence imaging of glycan uptake by intestinal bacteria. ISME J 13:1883-1889.Ziervogel et al., 2019
42. Reintjes G, Fuchs BM, Amann R, Arnosti C. 2020. Extensive Microbial Processing of Polysaccharides in the South Pacific Gyre via Selfish Uptake and Extracellular Hydrolysis. Front Microbiol 11:583158.
43. Balmonte JP, Hasler-Sheetal H, Glud RN, Andersen TJ, Sejr MK, Middelboe M, Teske A, Arnosti C. 2020. Sharp contrasts between freshwater and marine microbial enzymatic capabilities, community composition, and DOM pools in a NE Greenland fjord. Limnology and Oceanography 65:77-95.
44. Reintjes G, Fuchs BM, Scharfe M, Wiltshire KH, Amann R, Arnosti C. 2020. Short-term changes in polysaccharide utilization mechanisms of marine bacterioplankton during a spring phytoplankton bloom. Environmental Microbiology 22:1884-1900.
45. Ziervogel K, Arnosti C. 2020. Substantial Carbohydrate Hydrolase Activities in the Water Column of the Guaymas Basin (Gulf of California). Frontiers in Marine Science 6.
46. Balmonte JP, Simon M, Giebel H-A, Arnosti C. 2021. A sea change in microbial enzymes: Heterogeneous latitudinal and depth-related gradients in bulk water and particle-associated enzymatic activities from 30°S to 59°N in the Pacific Ocean. Limnology and Oceanography 66:3489-3507.
47. Klassen L, Reintjes G, Tingley JP, Jones DR, Hehemann JH, Smith AD, Schwinghamer TD, Arnosti C, Jin L, Alexander TW, Amundsen C, Thomas D, Amann R, McAllister TA, Abbott DW. 2021. Quantifying fluorescent glycan uptake to elucidate strain-level variability in foraging behaviors of rumen bacteria. Microbiome 9:23.2022
48. Brown SA, Balmonte JP, Hoarfrost A, Ghobrial S, Arnosti C. 2022. Depth-related patterns in microbial community responses to complex organic matter in the western North Atlantic Ocean. Biogeosciences 19:5617-5631.
49. Lloyd CC, Brown S, Balmonte JP, Hoarfrost A, Ghobrial S, Arnosti C. 2022. Particles act as ‘specialty centers’ with expanded enzymatic function throughout the water column in the western North Atlantic. Frontiers in Microbiology 13.
50. Manna V, Zoccarato L, Banchi E, Arnosti C, Grossart H-P, Celussi M. 2022. Linking lifestyle and foraging strategies of marine bacteria: selfish behaviour of particle-attached bacteria in the northern Adriatic Sea. Environmental Microbiology Reports 14:549-558.
51. Giljan G, Arnosti C, Kirstein IV, Amann R, Fuchs BM. 2022. Strong seasonal differences of bacterial polysaccharide utilization in the North Sea over an annual cycle. Environ Microbiol 24:2333-2347.
52. Robb CS, Hobbs JK, Pluvinage B, Reintjes G, Klassen L, Monteith S, Giljan G, Amundsen C, Vickers C, Hettle AG, Hills R, Nitin, Xing X, Montina T, Zandberg WF, Abbott DW, Boraston AB. 2022. Metabolism of a hybrid algal galactan by members of the human gut microbiome. Nature Chemical Biology 18:501-510.
53. Lloyd CC, Brown S, Balmonte JP, Hoarfrost A, Ghobrial S, Arnosti C. 2023. Links between regional and depth patterns of microbial communities and enzyme activities in the western North Atlantic Ocean. Marine Chemistry 255:104299.
54. Giljan G, Brown S, Lloyd CC, Ghobrial S, Amann R, Arnosti C. 2023. Selfish bacteria are active throughout the water column of the ocean. ISME Commun 3:11.
55. Brown S, Lloyd CC, Giljan G, Ghobrial S, Amann R, Arnosti C. 2024. Pulsed inputs of high molecular weight organic matter shift the mechanisms of substrate utilisation in marine bacterial communities. Environmental Microbiology 26:e16580.
56. Knittel K, Miksch S, Moncada C, Silva-Solar S, Moye J, Amann R, Arnosti C. 2024. Distinct actors drive different mechanisms of biopolymer processing in polar marine coastal sediments. Environmental Microbiology 26:e16687.
57. Ferrillo A, Tingley J, King M, Kidane A, Bajwa B, Xing X, Johannessen T, Lysberg A, Mydland L, Øverland M, Reintjes G, Shearer A, Klassen L, Low K, Patel T, Terry S, Pope P, Abbott DW, Hagen L. 2024. Microbial alginate foraging is conserved in geographically and taxonomically distinct ruminant microbiomes doi:10.1101/2024.12.17.628917.
58. King ML, Xing X, Reintjes G, Klassen L, Low KE, Alexander TW, Waldner M, Patel TR, Wade Abbott D. 2024. In vitro and ex vivo metabolism of chemically diverse fructans by bovine rumen Bifidobacterium and Lactobacillus species. Animal Microbiome 6:50.
59. Moncada C, Arnosti C, Brüwer JD, de Beer D, Amann R, Knittel K. 2024. Niche separation in bacterial communities and activities in porewater, loosely attached, and firmly attached fractions in permeable surface sediments. The ISME Journal 18:wrae159.
60. Balmonte JP, Giebel H-A, Arnosti C, Simon M, Wietz M. 2024. Distinct bacterial succession and functional response to alginate in the South, Equatorial, and North Pacific Ocean. Environmental Microbiology 26:e16594.•
61. Lloyd CC, Brown S, Giljan G, Ghobrial S, Vidal-Melgosa S, Steinke N, Hehemann JH, Amann R, Arnosti C. 2025. Interrelationships among carbohydrate inventories, enzyme activities, and microbial communities in the western North Atlantic Ocean. EGUsphere 2025-2249. doi.org/10.5194/egusphere-2025-2249
62. Čačković A, Pjevac P, Orlic S, Reintjes, G 2025. Selective heterotopic bacteria can selfishly process polysaccharides in freshwater lakes. Apr 22; 44(4) 115415. Doi: 10.1016/j.celrep.2025.115415
